# Next-Generation Internet of Things (IoT): Opportunities, Challenges, and Solutions

**DOI:** 10.3390/s21041174

**Published:** 2021-02-07

**Authors:** Yousaf Bin Zikria, Rashid Ali, Muhammad Khalil Afzal, Sung Won Kim

**Affiliations:** 1 Department of Information and Communication Engineering, Yeungnam University, Gyeungsan, Gyeungbuk 38541, Korea; yousafbinzikria@ynu.ac.kr; 2 School of Intelligent Mechatronics Engineering, Sejong University, Seoul 05006, Korea; rashidali@sejong.ac.kr; 3 COMSATS University, Islamabad, Wah Campus, Wah Cantt 47010, Pakistan; khalilafzal@ciitwah.edu.pk

It is predicted that by 2025, all devices will be connected to the Internet, subsequently causing the number of devices connected with the Internet to rise. As indicated by Cisco, there will be 500 billion devices connected with the Internet of Things (IoT) by 2030. Furthermore, Telefonica anticipates that 90% of vehicles will be associated with the IoT by 2030 and forecasts that every person will have an average of 15 connected devices by then. However, a review in 2015 proposed that more than 250 million connected vehicles will be available globally by 2020—an increase of 67%. The IoT offers numerous industrial opportunities that permit industries to create novel strategies and models to actualize their ideas. Such industrial opportunities additionally lead to effective and creative examination possibilities for researchers and specialists in multi-disciplinary research areas, thus combining research studies, engineering abilities, sciences and humanities all under one umbrella. Additionally, IoT is changing the world into a smart world in which everything is effectively accessible quickly. A few smart applications are shown in Figure 1. Generally, industries will engage in prompt speculation to deal with ventures depending on the development level of the IoT technologies. Simultaneously, IoT is causing our livelihood to flourish. These new technological advancements are making it conceivable to embrace this technology worldwide, while the user’s quality of experience (QoE) and applications’ quality of service (QoS) prerequisites are expanding radically. Several researchers have been consistently working to advance and develop new strategies to adapt to these phenomena. As shown in Figure 1, IoT applications are incredibly diverse and incorporate real-time multimedia, IoT-based healthcare systems, IoT-based next-generation smart industries and IoT-based next-generation smart agriculture. Besides security requirements, satisfying application necessities is a critical task; living in an epidemic situation requires fast data analysis and prediction mechanisms. Besides, the issues and challenges related to access technologies, such as spectrum scarcity, are another critical concern regarding the sharing of optimal resources between massive numbers of IoT devices. Utilizing AI-based solutions to enable dynamic and adaptive technologies is one of the potential ways forward in this area.

Consequently, this Special Issue has attracted researchers from academia and industries to investigate the opportunities for next-generation IoT-based user scenarios and study their effect on the solutions of the issues and challenges discussed above and propose viable solutions. Several researchers have contributed to different areas of interest related to next-generation IoT-based applications and user scenarios, including the following:

- Next-generation IoT-based smart healthcare;
- Next-generation IoT-based smart cities;
- Next-generation IoT-based smart agriculture;
- Next-generation IoT-based data analytics;
- Next-generation IoT-based industrial IoT;
- Next-generation IoT-based multimedia;
- Next-generation IoT-based spectrum sharing techniques.

Industrial IoT: The improvement of smart industries has had a vast and enduring effect on the inevitable future of global manufacturing. Industry 4.0 has consolidated smart industry into cyber-physical technologies, making intricate advancements more proficient and effective and improving the performance, quality, controllability, management and fairness of industrial measures in developing IoT-based smart industries. Next-generation cheaper sensing advancements are basic requirements for information collection and robust execution by manufacturing industries and supply chains. Simultaneously, a great deal of research has been conducted that has focused its attention on breaking down the industrial performance and usage of facilities. Most manufacturers need a profound understanding of the contrast between customary and pioneering manufacturing plant frameworks, as well as the wide arrangement of sensor advancements related to Industry 4.0. In [1], the authors highlight the distinctive available sensor technologies of Industry 4.0 and show the difference between conventional and smart industries. Moreover, the authors review the existing challenges that have been addressed in smart industry research and thus give an extensive overview of the related research on smart industries. The authors also summarize the variations between conventional and smart industrial facilities, outline the various sorts of sensors utilized in a smart factory and plan for future exploration, which includes the vigorous advancement of Industry 4.0 for smart industries.

Healthcare IoT: Some pathologies openly influence society, causing general healthcare issues. For example, pneumonic infections and chronic obstructive pulmonary disease (COPD) are presently the third leading cause of death, while tuberculosis is the ninth leading cause, with 1.7 million deaths and over 10.4 million further occurrences. Studies show that computational techniques, such as IoT-based technologies, contribute to health-related data analyses of lung pathologies by computerized tomography (CT). In [2], the authors propose another model reliant on IoT-based systems for the arrangement and division of pneumonic CT scans, applying the machine learning (ML) method for substantial learning strategies together with Parzen’s probability distribution. Their proposed model uses an application programming interface (API) considering the Internet of Medical Things to describe lung pictures. The approach was found to be efficient, with results above 98% precision when gathering aspiratory pictures. The model continued to the lung division stage, utilizing the Mask R-CNN organization method to make an aspiratory guide and use adjustment to locate the pneumonic fringes on the CT picture. The proposed strategy was performed in a better way than different works in the literature, arriving at high division measurement values; for example, an accuracy of 98.34% was achieved. In addition to the division time of 5.43 s and the improvement on other exchange learning models, their proposed process stands out among the others as it is entirely automatic.

Fog computing: Fog computing technology (FCT) recommends using computational resources that are close to the edge of the network. In [3], an FCT architecture is proposed for the deployment of a telemedical healthcare framework. The basic motivation for embracing the FCT-based framework in their proposed approach is to reduce latency and reliability. To prove the adequacy of their proposed approach in reducing reliability and latency, iFogSim-based simulations are compared with cloud-based networks. The outcomes show that a reduced latency and improved reliability can be accomplished by using the proposed approach to create a telemedical healthcare monitoring framework. In [4], the authors present a fog fragment computing technology (FFCT) solution that is compared with conventional FCT. The paper deals with information transmission using FCT edge nodes and their cooperation to overcome the extra required data transfer capacity for constrained IoT devices. The authors characterize the dynamic issue of the FCT using a ML approach and develop a Q-learning algorithm to achieve efficient decisions by driving the FCT edge nodes to help each other under special circumstances.

5G technologies: Current RFID tag technology is expensive and has not been exhibited as green technology for next-generation IoT in 5G advancements; furthermore, it is not sensible for long-range data transmission in 5G systems. A recently proposed I-RFID [5] tag uses the meandering angle technique (MAT) to assemble a design that satisfies the prerequisites of a less expensive printed antenna over the ultra-high-frequency (UHF) RFID band standard (860–960 MHz). In [5], RFID tags and MAT antennas are manufactured on a paper-based plate by screen and flexo-printing, which show less powerful results with a dielectric assortment as a result of mist and have a possible read range (RR) for European (EU; 866–868 MHz) and North American (NA; 902–928 MHz) UHF groups. Their proposed tag size was reduced by 36% to 38% compared to standard RFID tags; furthermore, the tag gain was improved by 23.6% to 33.12%, and its RR was enhanced by 50.9% and 59.6% for EU and NA UHF groups, respectively. It achieves impressive performance on some issues (plastic, paper and glass) and thus represents another state-of-the-art RFID tag with better attributes for 5G technologies. The future security trends for vehicular IoT (VIoT) in 5G technologies underline privacy assurance and security related issues utilizing a public keys framework. The essential concerns about proficient trust assessment, the malfunctioning of approved vehicles and secure data dissemination in vehicular wireless networks have not been investigated. To adapt to these difficulties, the authors of [6] propose a trust enhanced on-demand routing (TER) mechanism, which uses the TrustWalker (TW) algorithm for proficient trust evaluation and the route search method in VIoT. TER involved novel three-valued subjective logic (3VSL), TW measurements and the ad hoc on-demand distance vector (AODV) routing algorithm. This paper validates the precision of the proposed scheme in terms of its throughput, reliability and delay.

Network-on-a-chip (NoC): Recently, network-on-chip (NoC) technologies have become popular in communication frameworks for heterogeneous computing technologies as they are more scalable and highly efficient. Hostile technology scaling results in these designs having both permanent and transient issues regarding the chips. In [7], the authors point out the resilience of an NoC router to permanent flaws. A permanent flaw in an NoC router seriously impacts the performance of the whole network. The authors propose that the data transmission port can be utilized as a bypass method, for virtual channel (VC) queuing and for VC closing methodologies. The routing calculation step uses spatial redundancy and a twofold routing mechanism, and the VC allocation step uses spatial repetition. The switch allocation step uses run-time authority selection. Their proposed router is exceptionally fault-tolerant compared to the state-of-the-art NoC routers. The proposed mechanism achieves 7.98-fold higher reliability.

Next-generation IoT-based smart healthcare: The uses of IoT in healthcare are progressively being embraced in commercial domains. Several business solutions have been provided for the healthcare of an individual. However, there is enormous potential to enhance the IoT-based healthcare frameworks as an essential medical service and to develop hospitals into secondary healthcare units. In this way, it is profoundly significant to distinguish the expected advancements to address the issues and challenges resulting from accomplishing these objectives. The future perspectives include real-time location technology for Alzheimer’s patients who could wear a geospatial location sensor to allow their location to be tracked at any time. Other future technologies related to healthcare are as follows: the real-time detection of epileptic seizures and stroke, AR/VR-based tele-healthcare applications, handheld summarized healthcare records, blockchain-based secure services, remote surgery and remote interactive medical training.

Next-generation IoT-based smart-cities: The IoT system has advanced to develop certain arrangements in which each application is directly or indirectly linked with the context of the smart-cities application. Such frameworks and the collaborative applications can bring significant challenges and issues for IoT-based smart-cities systems. A few of the reliability challenges that have emerged in the IoT-based smart-cities framework are due to mobility issues in the transportation system; consequently, communication with vehicles is not reliable enough. Moreover, the presence of various sensor devices will cause some reliability difficulties regarding their failure. Some predetermined scenarios need communications between a massive number of smart devices, which are possibly disseminated at distinct geographical locations. The next-generation IoT frameworks are supposed to give a suitable platform that can investigate and incorporate the information emanating from various devices. Furthermore, the security and privacy aspects of such an IoT-based smart-cities system may be user-dependent information.

Next-generation IoT-based smart agriculture: Next-generation IoT-based smart agriculture is expected to assist with the advancements of farming practices and techniques to support sustainable farmers and resources. This is financially practical and sound, socially steady and performs well. This approach aims to maintain soil quality, reduce soil erosion and degradation and save water assets. A sustainable smart- agriculture system improves the biodiversity of the land and promotes a healthy and green environment. It is essential to coordinate this with the expanding interest into food and environmental change and the degradation of the biological system in the future. This has a significant role in safeguarding natural assets, reducing ozone-harming gas emissions, stopping biodiversity wastage and thinking about valued landscapes. The next generation IoT-based smart agriculture has been applied to cultivation to preserve natural resources without compromising the quality of the fundamental requirements. IoT-based smart agriculture’s common practices include smart farming for sustainable agriculture and smart crop rotations that mitigate issues and challenges related to weeds, plant disease, insects and other pests.

Next-generation IoT-based data analytics: With the advancement of IoT-based applications, there has been an extraordinary expansion in the number of connected devices continuously gathering data and interfacing with the environment in which they are embedded. Such smart devices empower the advancement of new data analytical techniques that are capable of making smart applications more intelligent and useful. Because of the collaboration between various applications, there is a tremendous amount of information from which helpful information can be separated. A significant issue that must be confronted is the recurrent occurrence of similar data generated by several connected smart devices. Embedding machine learning techniques, equipped with neural networks and deep learning algorithms, would help to tackle such issues and challenges. This kind of solution may require the extensive use of computing and energy resources. However, next-generation IoT-based data analytic solutions must be robust, cost-effective and use green technology.

Next-generation Industrial IoT: Industrial development has advanced significantly since the industrial technological revolution and has continued to grow due to IoT-based systems’ emergence. In fact, with the infusion and coordination of IoT-empowered sensors and actuators, smart manufacturing is changing customary product assembling processes, with numerous discrete industries working on the proof of concept and executing IoT advancements in their initiatives to drive benefits. However, industrialists are finding that these new IoT-based frameworks remain generally separated from their storehouses’ IT infrastructure and networks. Thus, industry is confronting different difficulties: complex cycles and sequential construction systems, network issues in assembling frameworks, unreliable distant access, security dangers, transparency and information standard models. To achieve the next-generation IoT-based industry, the integration of IT and operational technology is crucial for all manufacturing processes.

Next-generation IoT-based multimedia applications: Next-generation IoT-based multimedia applications also present several issues and challenges that must be carefully addressed. One of IoT’s primary goals—based on multimedia applications—is the provision of the maximum QoE and QoS in terms of reliability, throughput and extended network life-time. These objectives can be accomplished by utilizing proficient information aggregation, interoperable and sustainable architectures, effective feature extraction, smart routing, switching and intelligent MAC layer resource allocation. Moreover, there are limitations on IoT devices requiring them to be small and consume limited energy and computational resources. Thus, next-generation IoT-based multimedia applications require a redefined architecture to offer adaptability and interchangeability for numerous interactive media heterogeneous devices while thinking about the spectrum scarcity and resource constraints assets.

Next-generation IoT-based spectrum sharing techniques: It is assumed that the ever-extending IoT will create a large amount of data, leading to different prerequisites including throughput, reliability and latency. For example, a mobile device exploits existing base stations (BS) to gather information from enlisted IoT sensor devices and later forward this data to an edge server for additional information handling. Specifically, some particular IoT devices, which are at the edge of BS coverage, have rigid latency necessities on both uplink and downlink data transmission. However, due to the limited spectrum resources and the diverse nature of wireless networks, the requirements of next-generation IoT devices cannot be satisfied. Unfortunately, the current IoT technologies are not sufficient for this situation. To accomplish this, efficient AI-enabled resource sharing is required within the network. Indeed, spectrum sharing techniques between devices and networks need to be more adaptable and flexible.

Next-generation IoT-based security and privacy techniques: The consideration of the usefulness of IoT-based applications relies on how well they can handle security and privacy issues for trustworthiness. The problems and challenges regarding security and privacy that come with the current IoT may be critical in holding back the full reception of IoT. It is essential to realize that security and privacy privileges are key to achieving users’ confidence in IoT applications, connected devices and the related offered services. Several researchers have conducted work considering the security and privacy preservation in IoT-based systems. However, the key to the trustworthiness concerns is a direct result of the omnipresent AI-enabled frameworks, where the information handling and processing can be performed anywhere in the network. An AI-enabled network using Internet access is an essential feature that helps us to understand this issue because, unless there is an intelligent system architecture, it increases the ease of obtaining the individual user information.

Next-generation IoT-based cross-layer protocols: The new advancement and strategies in IoT-based protocols show that the network clustering methods provide adequate solutions to accomplish system effectiveness by selecting optimal routing cluster-heads. However, AI-enabled IoT-based devices face challenges due to several constraints, such as the low power of devices, a high user density, long-distance communication, higher latency and data losses. Cross-layer routing protocols are usually required for energy proficiency, lower latency and lower information transmission delay. Therefore, it is also critical to present AI-enabled cross-layer protocols for the next-generation IoT-based applications. This can be achieved with the help of several learning techniques, such as Reinforcement Learning. Such protocols must differ from the state-of-the-art protocols, mainly focusing on scalability and efficiency related to energy and QoS. Besides, it is required to have a cross-layer-based evaluation of IoT devices and select optimal and stable cluster heads using AI-enabled heuristic techniques with minimum overhead.

Recently, IoT has emerged as one of the most useful and fastest developing popular technologies. With the increase in the IoT’s popularity, users’ QoE and QoS requirements are increasing drastically. Researchers from academia and industry continuously need to improve and develop new procedures to adapt to these phenomena. Simultaneously, the IoT applications are incredibly different, incorporating smart-home, smart-healthcare, smart-industries, real-time multimedia and smart agriculture contexts. Thus, it is vital to deal with necessities, satisfaction and security issues. Utilizing AI-based solutions to make technologies robust and adaptive is another important task. Thus, in this Special Issue, we focus on presenting researchers who have investigated the potential uses of next-generation IoT-based solutions. Besides, we study the effect of next-generation IoT developments on the solutions to the challenges referenced above and propose viable solutions. This issue collects research articles covering different interest subjects that incorporate healthcare, smart-cities, smart-agriculture, IoT-based data analytics, smart-industries, IoT-based real-time multimedia applications, IoT-based spectrum sharing techniques, and IoT-based security and privacy techniques.

## Figures and Tables

**Figure 1:**
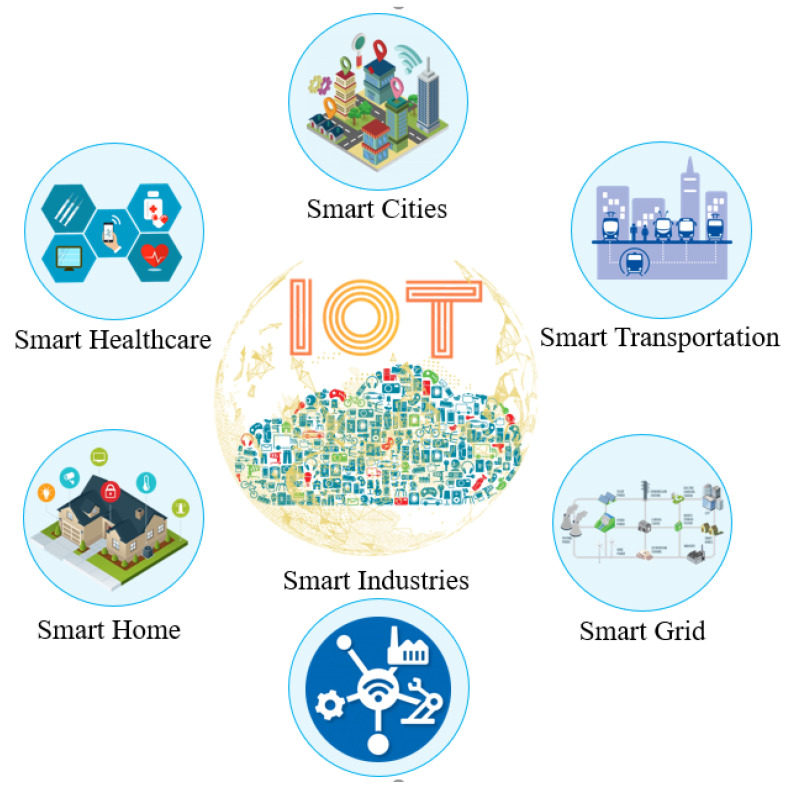
Next-generation Internet of Things (IoT)-based applications.

## References

[1] Kalsoom T., Ramzan N., Ahmed S., Ur-Rehman M. (2020). Advances in Sensor Technologies in the Era of Smart Factory and Industry 4.0. Sensors.

[2] Souza L.F.F., Silva I.C.L., Marques A.G., Silva F.H.S., Nunes V.X., Hassan M.M., Albuquerque V.H.C., Filho P.P.R. (2020). Internet of Medical Things: An Effective and Fully Automatic IoT Approach Using Deep Learning and Fine-Tuning to Lung CT Segmentation. Sensors.

[3] Hassan S.R., Ahmad I., Ahmad S., Alfaify A., Shafiq M. (2020). Remote Pain Monitoring Using Fog Computing for e-Healthcare: An Efficient Architecture. Sensors.

[4] Mobasheri M., Kim Y., Kim W. (2020). Fog Fragment Cooperation on Bandwidth Management Based on Reinforcement Learning. Sensors.

[5] Hussain M., Amin Y., Lee K.-G. (2020). A Compact and Flexible UHF RFID Tag Antenna for Massive IoT Devices in 5G System. Sensors.

[6] Sohail M., Ali R., Kashif M., Ali S., Mehta S., Zikria Y.B., Yu H. (2020). TrustWalker: An Efficient Trust Assessment in Vehicular Internet of Things (VIoT) with Security Consideration. Sensors.

[7] Rashid M., Baloch N.K., Shafique M.A., Hussain F., Saleem S., Zikria Y.B., Yu H. (2020). Fault-Tolerant Network-On-Chip Router Architecture Design for Heterogeneous Computing Systems in the Context of Internet of Things. Sensors.

